# Understanding regional variation in euthanasia using geomedical frameworks: a critical ethical reflection

**DOI:** 10.1007/s43999-023-00034-6

**Published:** 2023-11-29

**Authors:** A. Stef Groenewoud, Gert P. Westert, Theo A. Boer

**Affiliations:** 1 Scientific Center for Quality of Healthcare, Radboud University Medical Center, PO Box 9101, Nijmegen, 6500 HB the Netherlands; 2 Protestant Theological University, Groningen, the Netherlands; 3 University of Sunderland, Sunderland, England

**Keywords:** Euthanasia, Assisted dying, Geographical variation, Ethics, Practice variation, Preferences, Appropriate care, Supplier induced demand

## Abstract

Considerable geographical variation in the uptake of euthanasia has been reported: some municipalities in the Netherlands have a 25 times higher euthanasia rate than other municipalities. Current ‘geomedical’ frameworks for interpreting practice variation in health care utilization seem inadequately tailored to understand regional variation in morally controversial procedures such as euthanasia. The aim of this conceptual article is threefold: i) to add relevant medical ethical principles to current frameworks; ii) to provide a four-step ethical-geomedical model for the interpretation of geographical differences in the utilization of health care in general and for ethically controversial treatments in specific; iii) to gain better understanding of the existing geographical variation in the incidence of euthanasia by using this framework in our analysis.

**Supplementary Information:**

The online version contains supplementary material available at 10.1007/s43999-023-00034-6.

## Introduction

It is well-known that quality, utilization and costs of end of life (EoL) health care vary between regions^1^ [1]. The Atlas of Variation in Palliative and End of Life Care for England shows that on average 22.8% of deaths occurred at home, with a variation by Clinical Commissioning Group (CCG)^2^ of between 18.2% and 30.1%, a 1.7-fold difference [2]. The Dartmouth Atlas of Health Care shows that in the United States the number of hospital admissions per decedent during the last six months of their lives varies per hospital service area between 0,57 and 2,67, a factor 4,68 difference [3]. “*Where you live is how you die*,” was the conclusion of the researchers [4]. In both instances, the focus is on the signaling of variation rather than on its explanation. Both atlases recommend that differences should be interpreted in the light of demographic data and of the need and availability of specialist palliative care. They do not explore to what extent these instances may display unwarranted practice variation.

Regional variation in the uptake of euthanasia and physician assisted suicide was first addressed by Koopman and Putter. However, they had only access to data on the level of the five Dutch euthanasia regions [5]. In our recent study of regional variation in euthanasia we distinguished between 90 regions, 326 municipalities within these regions, and thousands of districts within these municipalities [6]. We found that the incidence of euthanasia as a percentage of all deaths is more than 25 times higher in municipalities with the highest rates than in municipalities with the lowest euthanasia rates. Part of the variation is associated with age, church attendance, political orientation, income, self-experienced health and availability of voluntary workers. After adjustment for these characteristics a considerable amount of geographical variation remained (factor 7). This calls for further exploration.

From our attempts to understand and interpret this significant regional variation in euthanasia, and from the responses that followed our publication [7], we learned that appropriate interpretation of regional variation in euthanasia is not easy for two reasons. First, current frameworks for the interpretation of regional variation in health care focus on regular health care activities only. Euthanasia, despite being a legal practice in some countries,^3^ is still considered an exceptional medical procedure [8]. Second, existing frameworks for interpreting practice variation include mainly medical, epidemiological and at most sociological elements and lack ethical weighting and reflection. This omission is not only problematic because regional variation is usually denoted in terms of ‘warranted’ or ‘unwarranted,’ which is in itself a value laden terminology, but statements such as “geography is destiny”^4^ or: “where you live is how you die” [9] are all the more morally debatable in the case of ethically controversial ways to die.

This article’s aim is threefold. First we want to deepen our understanding of regional euthanasia variation, using existing and widely accepted frameworks. Second, we suggest to broaden the scope of those frameworks by including relevant ethical aspects in our analysis of regional euthanasia variation. Third, we believe that the inclusion of ethical aspects will also be helpful in understanding regional variation in the utilization of other forms of healthcare (diagnosis, treatment, etc.).

In the sections below we 1) summarize current theoretical, geomedical frameworks for the interpretation of regional variation in health care^5^ and expand them with medical ethical principles that have become known as the principalist account^6^ [10]. Then we 2) use the new framework to analyze and interpret findings^7^ from recently published research in regional variation in the incidence of euthanasia. Finally, we 3) conclude on what can be done next in terms of future research as well as quality improvement.

### Geomedical frameworks for the interpretation of regional variation in health care utilization

#### Warranted and unwarranted variation

**Fig. 1:**
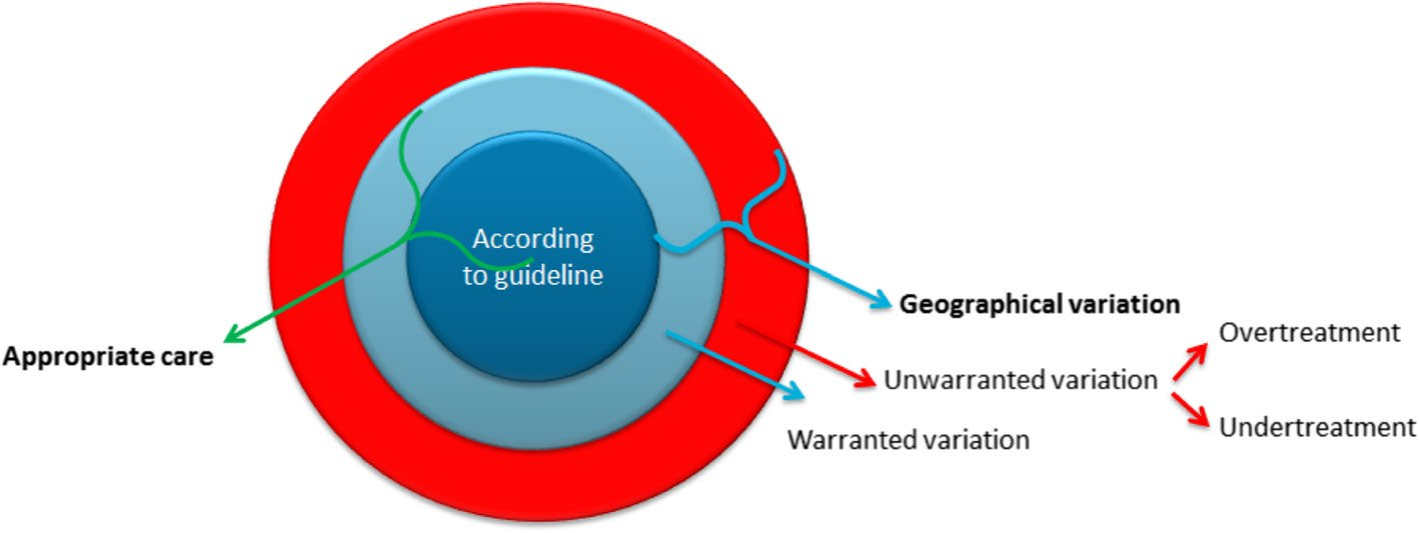
A vocabulary of variation

The ultimate challenge for the right interpretation of regional variation in health care quality, utilization, and costs is discerning between warranted and unwarranted variation. Over the last decades, awareness has grown that part of the observed variations may exist for good reasons, and that only the unwarranted variation should be minimized [11, 12]. According to Wennberg, one of the founding fathers of research in regional variation in health care, regional variation is acceptable only if it can be explained in terms of specific regional pathologies – e.g., specific types of cancer with regional causes – or in terms of differences in patient preferences – e.g., women’s preferences to give birth either at home or in a clinical setting [11, 12]. Variation for other reasons should most likely be classified as either overtreatment or undertreatment (see Fig. 1) [13].

Whereas Wennberg’s deductive method leads to a residual category of ‘unwarranted variation’ which is not further elaborated, Mercuri and Gafni made an inventory and an appraisal of theoretical frameworks for evaluating unwarranted variation [14]. They found three additional explicit frameworks that specify forms and causes of unwarranted variation: i) Goodman stated that practice variation is unwarranted if caused by insufficient quality, inappropriate care, or lacking efficiency of healthcare [15]. This might e.g. be the case if in some areas patients are deprived of appropriate care because of long waiting lists. ii) For Bojakovski, unwarranted is that part of regional variation that cannot be explained in terms of public needs or medical needs or preferences, and that exists despite compelling evidence or agreement among providers as to the (evidence-based) best course of treatment [16]. iii) Sepucha et al. state that there are multiple sources of unwarranted variation including inequitable access to resources, poor communication, role confusion, and the misinterpretation or misapplication of the relevant clinical evidence [17]. More recent publications, for example by Birkmeyer et al. [18] focus on finding evidence for these explanations, and on finding ways to enhance appropriateness of care, such as shared decision making. A fourth theory, called a *theory of local standards*, was developed by Westert and Groenewegen in the 1990s [19]. Instead of explaining regional variation (which they perceive as a normal phenomenon due to professional uncertainty), they focus on understanding homogeneity in physicians’ local practices in certain areas. They mention several possible explanatory *social* factors such as financial and organizational enablers and constraints, but also physicians’ natural inclination to search for social approval of colleagues (‘stay with the pack’). This alternative approach was further developed by De Jong, who discerned three groups of factors that may explain local homogeneity in health care practices: selection (residing physicians select new colleagues who think and act likewise), gradual adaptation to local norms, and rapid adaptation to circumstances such as financial and regulative opportunities and barriers [20].

These four proposals bring thus more detail and depth to the three layers of Wennberg’s model. Most of the factors mentioned (e.g., by Goodman and by Westert) are variants of what Wennberg calls supply-induced, or demand-side specifications (patient preferences). We will therefore retain this threefold division of Wennberg in the sequel, but keep in mind the differentiations made by the other four theories.

In a further attempt to understand geographical variation in health care, Wennberg introduced a distinction between three categories of care, each with their own criteria for the (un)warrantedness of variation: i) effective or necessary care is health care which – according to current medical evidence – should *always* be given to eligible patients. ii) preference-sensitive care is care which application may vary according to patients’ preferences. iii) supply-sensitive care which frequency and intensity of use is highly determined by the medical professionals’ decisions (see textbox 1 in Supplementary Materials for more details) [11, 12, 21].

#### Adding core medical ethical principles

These approaches for understanding and explaining regional variation in health care use contain only medical (evidence), epidemiological (health differences) and sociological (preferences) elements. To our knowledge hardly any literature exists that discusses ethical issues of regional variation in health care utilization in general, and even fewer in EoL care in specific. In 2001, *JME* published a paper that discusses equity issues in countries with regionally organized systems of health care financing and insurance [22]. We found only one paper that ethically reflects on regional variation in health care utilization at the EoL [23]. In a clear allusion to Beauchamp’s and Childress’ four principles of medical ethics^8^ [10] that article discusses three ethical issues regarding geographical variation in healthcare utilization at the EoL. First, regional variation undermines *distributive justice* because benefits (use) and burdens (taxes and insurance premiums) are shared inequitably. Second, regional variation may violate the principle of *nonmaleficence*, e.g., when patients receive ineffective or avoidable interventions, or when tax payers have to contribute to funding low value care or even waste. Third, regional differences in quality, costs, and utilization of EoL care should be communicated to the public in order to facilitate informed choices and to prevent infringements upon the principle of *autonomy*. (See textbox 1 for more details on these principles).

#### Integrated model for the interpretation of (un)warranted regional variation in euthanasia

**Fig. 2:**
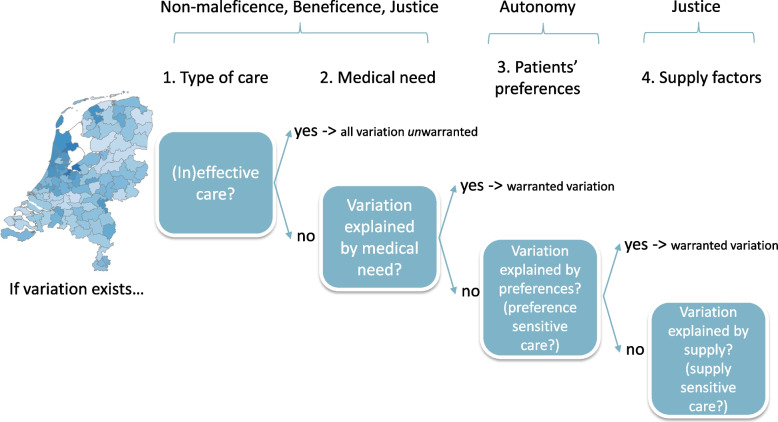
Integrated model for the interpretation of regional variation in the incidence of euthanasia

Our next step would be to line current geomedical models for understanding (un)warranted variation up with the above mentioned medical ethical principles (see Fig. 2). We believe that the questions about ‘type of care’ and about ‘differences in need of care’ roughly coincide with the principles of beneficence and non-maleficence, the questions about preferences with the principle of autonomy and the questions about supply with the principle of justice. This is of course a rough typology; in practice, per step several ethical principles (also different ones than those mentioned so far) may play a role. In the analysis below, we apply the model to the phenomenon of geographical variation in the incidence of euthanasia in the Netherlands, however – because it consists of general geomedical and ethical principles – we believe it is equally applicable to *all* forms of health care delivery. Note that the four-step framework is intended for the step-by-step interpretation and interpretation of geographic differences at the *population level*. If we were to consider the appropriateness of care at the individual level, a holistic approach, involving all four layers simultaneously would be more appropriate.

### Analysis

In our research of practice variation in euthanasia (details in text box 3 in Supplementary Materials), we found significant differences between low- and high-incidence regions, municipalities, and districts [6]. On a municipality level, a factor score of 25.28 was found, which means that in some municipalities euthanasia occurs up to 25.28 times more frequently than in other districts. (Importantly, we signalled a slight decrease in practice variation.) We interpreted this variation in the incidence of euthanasia using Wennberg’s deductive model [11, 12]. For 17 variables the possible association with the variation found was considered. Six of these – age, church attendance, political affiliation, availability of volunteers, income, and self-perceived health – appeared to be statistically significant associated. These variables were either related to health differences (step 1 Wennberg model), or to patient preferences (step 2). To some degree, the remaining and unexplained variation may have been caused by supply factors, such as the presence or absence of good alternatives, or physicians’ preparedness to perform euthanasia. Since this could, in Wennberg’s terms, be indicative of some degree of unwarranted variation in the uptake of euthanasia, we will in this article focus on possible factors beyond pathologies and patient preferences, and will do so by using the integrated interpretative model described above (Fig. 2). For each step, we discuss to what extent it applies to the act of euthanasia and discuss what can be said from the perspective of medical ethics about, and possibly added to, Wennberg’s model.

#### Step 1: Euthanasia: (in)effective care?

We think that euthanasia can hardly be characterized as ‘effective care’ in the Wennbergian sense that it must always be given under certain conditions, and that otherwise the patient would be undertreated. We name three arguments, all taken from the Netherlands, which has the oldest and broadest euthanasia practice worldwide. First, terminating a patient’s life continues to be a legally punishable act for which the doctor can only be acquitted if a number of criteria – a patient request and the presence of unbearable and irremediable suffering being the most important – has been met [24]. Secondly, regardless of the legal regime, euthanasia is still widely considered as a ‘transgressive’ act, only to be considered if no other options are left [25]. In the Royal Dutch Medical Association’s recent report *Decisions at the End of Life* euthanasia continues to feature as a last resort at the end of a trajectory of Advance Care Planning, good palliative care, avoidance of medically futile treatments, and advance directives, and in which alternatives such as the voluntary stopping of eating and drinking and assisted suicide are considered [26]. Thirdly, the reported practice variation (and the virtual absence of euthanasia in some Dutch areas) indicates that euthanasia is still not an integrated procedure in medical practice. In terms of Rogers’ diffusion theory [27]: although the incidence of euthanasia in traditionally low-incidence areas increases over time, its ‘diffusion,’ possibly because of its transgressive character, still seems to be slower than for normal medical interventions.^9^

On the other hand, the conclusion that euthanasia is always *in*effective, i.e., that it should never be given regardless of the situation, is equally hard to uphold. First and foremost, we would have to ignore or declassify the legal, ethical, and professional reasons for which several countries decided to allow euthanasia. Second, in countries where it is possible, both the numbers and the pathologies expand rapidly [28, 29]. In practice, this has increased the professional uncertainty, the ‘grey zone’ surrounding the ‘appropriate indication,’ and has left more room for interpretation of the due care criteria in individual cases. On the other hand, given the aforementioned transgressive character of euthanasia and the fact that it is a last resort option, we suggest that high euthanasia rates in certain regions justify further investigation of the euthanasia practice in such regions and a quest for possible causes. ‘Desired’ does not always mean ‘desirable,’ so questions must continue to be asked to what extent the proliferation of euthanasia is indicative of its quality, effectiveness, and need. We therefore conclude that a discourse in terms of effective care and ineffective care is problematic. Describing euthanasia in terms of “a *service that, on the basis of reasonably sound medical evidence should always be given because it, is known to work better than any alternative,”* may even strike many people as repulsive.

Even more problematic is a discourse in which euthanasia is characterized in terms of its efficiency or inefficiency [30]. In a recent paper, David Shaw and Alex Morton advocate the inclusion of economic arguments for the possibility of euthanasia [31]. They argue that “*the cumulative avoidance of negative quality-adjusted life years”* that may be the result of allowing euthanasia, will benefit both individuals and societies. Efficiency considerations, however, are misplaced for several reasons. If we conclude that euthanasia is the most ‘efficient’ option for a patient and their society, why not advocate euthanasia without the patient’s request? On a philosophical level, it is problematic whether we can at all make comparisons in terms of ‘negative quality-adjusted life years.’ Is it at all possible to claim that ‘it is better for someone to be dead than alive’? In normal choices about efficiency of medical procedures, comparisons are made between one effect of a procedure and another; in both cases it is about the quality of life of living patients. Comparing quality of life and quality of not-living is a different matter. We therefore preferably talk about the (in)appropriateness of euthanasia in terms of ‘non-maleficence’ and ‘beneficence’ (note the order) and not in terms of ‘(in)effective’ or ‘(in)efficient’ care. We think the original definition of evidence based medicine, that takes the individual patient’s values as a starting point for evidence based treatment, [32] provides good guidance for appropriate end-of-life care but will not necessarily reduce variation.

#### Step 2: variation explained by differences in medical need?

Second, geographical variation in the utilization of care might be warranted if it is caused by differences in medical need. In other words: a higher proportion of euthanasia could be justified in high incidence regions, if more patients experience unbearable suffering without prospect of improvement in those regions. Since ‘unbearable’ is a highly subjective concept and no criteria for gauging unbearable suffering exist, no data are available about possible regional differences that could help us explain regional variation in the uptake of euthanasia. A proxy for this might be to ask whether the observed variation is linked to differences in the prevalence of certain *diseases* and in the population’s *health*. In our study we therefore included several health-related variables in our regression model: the percentage of inhabitants who felt depressed; the percentage of inhabitants with functional, physical, visual or auditory limitations; the percentage of inhabitants who had good self-management, and who were in good health. Secondly, we took the incidence of euthanasia as a percentage of the total number of deaths per area. The idea was that if the high incidence of euthanasia in some areas is associated with a higher incidence of, for example, cancer and, consequently, a higher prevalence of terminally ill cancer patients, then our standardization to numbers of deaths would already take this into account.^10^

In our analyses, the euthanasia rate was indeed associated with a population’s self-perceived health. Strikingly, this association turned out to different from what one would intuitively assume: a higher self-perceived health turned out to be associated with a higher euthanasia rate. Being aware of the risk of the ecological fallacy, we nevertheless also hypothesize that some people may be more inclined to request euthanasia when their own poor health contrasts sharply with the average better health in their immediate surroundings, similar to when poverty is experienced as harder for people living in affluent neighborhoods. From a medical ethical point of view, Wennberg’s assumption that geographical differences in health care utilization are justified by geographical differences in health is understandable because it is in line with the principle of beneficence for the most vulnerable that is normative in (strong) egalitarian societies. This is however not self-evident. Other mechanisms for the distribution of scarce resources in health care also occur. Firstly, in liberal, utilitarian societies, where healthcare is considered a free market, criteria such as merit, status or contribution also occur as principles of distribution [33]. The geographical distribution of e.g. (un)*employment* or *income* can therefore also be a variable to take into account, as we did in our study. In our model, (un)employment was not found to be statistically significantly associated with the incidence of euthanasia. However, a higher average income in a municipality was found to be associated with a higher euthanasia rate. The moral question rises whether this is desirable: both from the principle of ‘equal access to care,’ and also as a cultural phenomenon. What if richer people tend to have a death wish more easily than the less well-to-do?

Another distribution mechanism in the face of scarce medical resources is, as Daniel Callahan has argued, to distribute care on the basis of age. Callahan proposes to limit the use of ‘acute care’ and to expand ‘comfort care’ for elderly people who have already completed a normal ‘natural life span’ [34]. Then what would such age-based rationing mean for the distribution of euthanasia: that people more eligible for receiving euthanasia solely for being aged, irrespective of their medical needs? [33]. For this reason we also included the variable ‘age’ in our model of analyzing regional variation of euthanasia. Contrary to what some might expect, not old-age, but ‘middle age’ – i.e., a relatively large percentage of people aged 45–65 – was statistically significantly associated with a higher incidence of euthanasia.

But even the concept of ‘medical need’ as used by Wennberg is as not as unambiguous as it seems. Bioethicist Yvonne Denier warns that what once featured as conditions that belong to life have become so medicalized that they lead to ‘medical need.’ This ‘needs-inflation’ leads to “*opportunistic hijacking of social resources”* [35]. Others have also linked geographical variation in health care utilization to the phenomenon of ‘medicalization’ of our societies [36]. Denier makes two proposals for further defining people’s (medical) needs: one is based on what John Rawls and Norman Daniels call ‘primary goods’: *“things that persons need in their status as free and equal citizens, and as normal and fully cooperating members of society over a complete life.”* The other is based on Martha Nussbaum’s capability approach, and focuses on “*the generic characteristics of human existence, which encompasses both dependence and independence as typical features of human life*.” [ibid.]. An interesting exercise – for which this article unfortunately lacks space – would be to use such alternative approaches, taking into account the ever-increasing medicalization of our existence, to reflect on the question of what ‘(medical) need’ euthanasia is an answer to. In a terminally ill patient in unbearable suffering this may be more obvious than patients with a much longer life-expectancy. Addressing these questions could be helpful in the second step of Wennberg’s interpretative model.

In our study, after having statistically adjusted for, among other things, self-perceived health, income and age, a considerable unexplained variation still remained. This forces us to look at another possible explanation: the geographical differences in people’s preferences.

#### Step 3: variation explained by differences in patients’ preferences?

If we are not dealing with effective or ineffective (and efficient or inefficient) care (step 1) and if variation remains after adjusting for relevant disease and health-related factors (step 2), perhaps (part of) the variation can be explained by geographic differences in patient preferences. In our study we therefore included as many variables as possible in our initial statistical model that are related to patients’ preferences, and for which data was available at the level of all Dutch municipalities. Our final regression model contained seven of such variables: i) the percentage of votes for political parties in the 2017 parliamentary elections, divided into conservative parties, middle parties and liberal parties; ii) the percentage of inhabitants who reported church attendance at least once per month; iii) the percentage of inhabitants’ self-reported religious beliefs; iv) the percentage of non-Western immigrants; v) the composition of households; vi) being unemployed or incapacitated; vii) median annual income. Three of these appeared to be statistically significantly associated with the percentage of euthanasia: church attendance, political orientation, and income.^11^

Morally, it is not without question that euthanasia can be characterized as ‘preferences sensitive care’^12^ and that an important indicator whether geographical variation is warranted, is that corresponds to a variation in patient preferences. In the third step of Wennberg’s explanatory model autonomy seems to be dominant indeed. But what kind of autonomy? Beauchamp and Childress (see textbox 2, Supplementary Materials) distinguish between autonomy as *agency*, i.e., a personal capacity to make a well-informed and deliberate choice and autonomy as *freedom*, i.e., as a right to have one’s choice respected or carried out [10]. These concepts are further refined by Schermer, when in addition to autonomy as a capacity and autonomy as a right she suggests that autonomy may be a *virtuous condition*^13^ [40]. Joel Anderson adds another relevant element, namely that under different societal ‘regimes’ (neo liberal, solidaristic or perfectionist) autonomy may mean different things [41].

We will now first elaborate on the three meanings of autonomy as suggested by Schermer, and their relevance in understanding regional variation in euthanasia. *Autonomy as capacity* refers to the fact that an agent must have a certain capacity, encompassing both free will and rationality. Rationality presupposes that the autonomous agent is well aware of the nature and consequences of a desired action. One could therefore say that the most radical decision a human being can make, namely ending one’s life and paradoxically giving up one’s autonomy, puts the highest imaginable demands on the capacities of the agent. However, research shows that a considerable part of Dutch citizens have limited knowledge of euthanasia practice and its surrounding regulations. In addition, nearly half of respondents responded that they did not know what palliative care entails [37, 38]. Although physicians will help patients to mend possible deficiencies and to make a well-informed and free decision, their vulnerability and physical limitations may have adverse effects. This is a special point of attention for patients with diminished cognitive abilities and even in incapacitated patients^14^ [28]. Recent studies in euthanasia in dementia for example show that hardly any specific attention is given to tailor made information for these patients [42, 43].

As for *autonomy as a right* – one’s sovereign right to make and act upon one’s own choices without being interfered, involuntary influenced or forced by others [40] – we believe that also this dimension of autonomy is not fully uncontested in the sphere of euthanasia. First, euthanasia is hardly anywhere a patient’s right, and in the end it is the doctor who decides to grant a request or not. Moreover, few patients make decisions in complete isolation: they are tied in social relationships and form their opinions about a dignified death in the context of evolving societal norms which, when it comes to the Netherlands, include an increasing normalcy of euthanasia [39]. Difficult experiences with problematic medical decisions at the end of life of one’s loved ones (‘ghosts from the past’) influence one’s own expectations, fears, and preferences [42]. As for one’s social relationships, it is well-documented that *“feeling a burden to others is one of the primary triggers of the wish to hasten death”* [44, 45]. The prevalence of such feelings may regionally vary as well. This was also illustrated in our data since our initial regression models showed that ‘loneliness’ was statistically associated with differences in the incidence of euthanasia. After discussion in our team, we decided not to adjust for this variable. Justification of a relatively high incidence of euthanasia because of higher percentages of loneliness in certain regions would be similar to adjusting the incidence of lung cancer for the percentage of smokers in an area. Just as in that case we do not accept the large number of smokers (that has to be brought down with quit-smoking campaigns), communities should instead be committed to reducing loneliness within their ranks. Adjusting for these kinds of factors would rather disguise the urgency of this and is therefore undesirable.

*Autonomy as a virtuous character trait* encompasses a combination of several virtues: self-possession, individuality, authenticity, self-legislation, self-determination, initiative, and responsibility for self. At the same time humans are ‘social animals’ whose autonomous choices must be consistent with their membership of a community [40]. Portraying a euthanasia request as a predominantly individual choice would thus be problematic in two ways. First, we would ignore its societal impact, e.g., the effect that one patient’s choice for an assisted death may have for the choices and paths of their loved ones. This phenomenon – including a declining solidarity with those who value the ‘giftedness of life’ – has been described before in the sphere of pre implantation genetic diagnostics, another field in which one such choice may lead to others considering to make a similar choice [46]. Second, an emphasis on the individual choice for euthanasia ignores the fact that this choice also has important implications on the emotions of their loved ones. A description of 44 stories of experience of relatives of patients who opted for euthanasia shows that for relatives there are important downsides to this choice [47].

Finally, and with Joel Anderson, we must acknowledge that there are different *‘regimes of autonomy’:* neoliberal, solidaristic, and perfectionist. These regimes entail conflicting understandings of what gets you autonomy and what autonomy gets you. For a neo-liberal approach, what is central is that what gets you autonomy is minimal and what autonomy gets you is maximal [41]. Perhaps the claim that euthanasia is preferences-sensitive care and that geographical variation can be fully explained and justified by regional differences in preferences, does fit within neoliberal spheres. This may be different however in more solidaristic communities. If, as we discussed above, not all members of society have the same capacities to make end-of-life-care choices, and in the absence of equal access to appropriate end of life care, then a solidaristic society may even limit choice options instead of enlarging them: *“a solidaristic regime is committed to minimizing the effects of (…) of lower autonomy-competence (and the comparative advantage to those with higher forms of competence), and one likely way of doing this is to limit the situations of choice that require these forms of competence. Thus, by limiting the range of options — and perhaps even refusing to license anyone to request physician assisted suicide — a solidaristic regime aims to protect the vulnerable and minimize inequality.”* [Ibid.] Perfectionists share the solidarists’ concern with individuals’ autonomy-competencies being insufficient to handle the expanded context of end-of-life options, particularly when that includes physician-assisted suicide. But perfectionist regimes take a different approach to rectifying this, focus not on a restriction of choice but an expansion of the skills needed to handle the expanded choice [ibid].

After elaborating on the key concept of autonomy, we come to the intermediate conclusion that – because of its conceptual and ethical ambiguity – patients’ preferences fail to fully and morally justifiably explain regional variation in the incidence euthanasia, even if it would do so in a statistical analysis.

#### Step 4: supplier induced demand?

After we adjusted for health related and preferential factors (steps 1–3), we reported a remaining variation of about a factor 10 between high- and low-incidence municipalities. Although we cannot rule out that other demand-side factors play a role than those we included in our model (think e.g. of death anxiety ( both by patients and physicians), anticipated suffering, psychological binding types), we still think we can justifiably hypothesize that at least part of the remaining variation is directly or indirectly related to differences in the *supply* of end-of-life care.

*Direct* supplier induced demand in euthanasia exists if the incidence of euthanasia is influenced by a doctor’s preparedness and inclination or aversion to supply euthanasia. Doctors’ influence on EoL decisions, catalyzed by their own views on appropriate care, has been described in the literature before [48, 49]. *Indirect* supplier induced demand happens when the incidence of euthanasia is influenced by the access to, the availability and the quality of alternative EoL-options, for example if patients would request euthanasia because of the absence of (good) palliative care, or assistance in the voluntary stopping with eating and drinking. Unfortunately, though there is a European atlas of palliative care [50], no such data exists on the availability and accessibility of palliative care in Dutch municipalities. A survey among SCEN-doctors (legally required second-opinion doctors prior to a euthanasia), showed that suboptimal palliative care was a factor in 10% of euthanasia requests [51, 52].

In the absence of data on the level of municipalities or districts (and despite the existence of a national quality framework for palliative care in the Netherlands [53]) we included some proxy variables in our data, such as ‘travel distance to the nearest hospital,’ ‘availability of informal care,’ and ‘the availability of voluntary workers in a municipality.’ The latter variable was chosen as a proxy for social cohesion in a municipality, and appeared to be statistically significant related with the incidence of euthanasia. This immediately raises the question if adjustment for this variable is necessary (i.e. less voluntary workers in a community makes a higher percentage of euthanasia more understandable and warranted) or – the opposite – that adjustment for such variables would blur our understanding of geographical patterns in the incidence of euthanasia (i.e. the shortage of voluntary workers is something municipalities should work on, instead of being compensated for).

What else could be said about the moral justifiability or reprehensibility of supplier induced demand in euthanasia? On the one hand, one could reason that it is completely defendable that doctors influence patients’ choices at the end of life. As in so many medical procedures, not all patients are able or willing to make informed choices all by themselves. For many, *shared decision making* – including the active involvement of a doctor in making decisions – features as a successful strategy to reduce inappropriate care and unwarranted variation [54]. On the other hand, doctors may also be biased, and, as a consequence, have unjustified, unwarranted impact on their patients’ choices. In one of the scarce bioethical papers on geographical variation, Weeks and Nelson describe how supply-driven geographical variation causes infringements upon three principles [23]. First, upon the principle of justice: patients have unequal access to EoL- care and collective resources may be improperly used. Second, upon the principle of non-maleficence: patients may suffer from both undertreatment and overtreatment. Third, upon patients’ autonomy: how should they inform themselves about medical care at the EoL that is not available or accessible?

Where does this analysis leave us? What conclusions can be drawn after having introduced and applied our ethical geomedical model for the analysis of geographical variation in health care utilization?

## Conclusion

This paper had three aims: i) to understand and interpret the regional variation in the incidence of euthanasia; ii) to take a broader view than current geomedical frameworks for the interpretation of geographical variation usually do, by adding some relevant medical ethical principles; iii) to provide a combined ethical, geomedical model for the future interpretation of geographical differences in the utilization of health care.

The main added value of the paper is that we have shown that a) people’s subjective trade-offs become part of regional patterns of care use, in this case euthanasia; b) that it matters to add moral principles to the originally neoliberal-based explanatory models of geographical variation in care use. Regional differences cannot be explained and justified purely on the basis of objectively identifiable differences in care needs, preferences and supply factors.

In addition, we must acknowledge that – in the analyses we made in our earlier work, in order to explain step by step the variation in the incidence of euthanasia – in the choice of variables we were dependent of the availability of data at the regional, municipal and district levels. We do not rule out the possibility that due to this limitation, part of the variation remained unexplained. At the same time, this underscores the importance of looking beyond health, preferential, and supply-factors when analyzing and interpreting geographic variation in health care. Even if these factors were perfectly measurable and translatable into statistical models, it is still necessary to include moral considerations in interpreting this variation.

Our integrated, deductive ethical geomedical model for the interpretation of regional variation in euthanasia combines existing geomedical models with relevant ethical principles, such as beneficence, non-maleficence, autonomy, and justice. By placing them in a decision-tree-format, we enable researchers in future projects to systematically discuss about, and discern between unwarranted and warranted variation.

The combination of ethical principles with geomedical variables provided additional dimensions, and useful language for discussing and understanding geographical variation. For example: in the first step we learned that it is impossible to define euthanasia (as well as other medical decisions at the EoL) as ‘(in)effective care.’ Preferably we talk about the ‘appropriateness’ of such choices which should be discussed from case to case. We conclude that – since euthanasia is not a form of ‘regular medicine’, it is hard to use classical geomedical frameworks that were originally developed for elective procedures in their original form. Probably, we should even choose not to use the terms ‘warranted’ and ‘unwarranted’ in the sphere of euthanasia. From our second step it became clear that ‘medical need’ is a multi-layered concept that requires discussion before it can be decided to what degree it explains geographical variation. It also raises new moral questions, for example: to what (medical) need euthanasia is presented as a solution. Third, we found that it is not self-evident that patients’ preferences may completely justify geographical variation in health care utilization. Different conceptions of autonomy and its embedding in different societies require careful reconstruction before it can be decided if variation in preferences makes variation in health care utilization warranted. Fourth, we learned that not in all cases supplier induced demand is bad. On the other hand, we hypothesize that in some areas there is a different ‘local standard’ regarding medical decisions at the EoL.

Finally, it is important to note that existing geomedical models are purely data-driven. If we really want to understand the 'couleur locale' of healthcare practice (in this case: euthanasia practice) in a region, it is important not only to measure preferences through all kinds of data sets, but also to enter into dialogue with patients, their loved ones and with the healthcare providers involved. We strongly recommend that in the future such qualitative research be part of the interpretation and interpretation of geographic variation in healthcare use.

All in all we believe that our model is not only useful for medical decisions at the EoL, but for all medical treatments and therapies whose utilization, quality and costs vary between regions.

### Supplementary Information

**Additional file 1.**

**Additional file 2.**

## Data Availability

Data are available upon reasonable request.

## Footnotes

1 In this paper we interchangeably use the concepts of regional variation, geographical variation and practice variation.

2 Clinical Commissioning Groups (CCGs) were created in the UK following the Health and Social Care Act in 2012, and replaced Primary Care Trusts on 1 April 2013. They are clinically-led statutory NHS bodies responsible for the planning and commissioning of health care services for their local area. As of 1 April 2019 there are 191 CCGs in England. https://www.nhscc.org/ccgs/

3 ‘Euthanasia is generally defined as the intentional ending of a patient’s life at their explicit request, by a physician. As for the legal status of euthanasia and assisted suicide, the situation in 2023 is described in the supplementary material, Table 1.

4 The phrase “Geography is Destiny” originally stems from geopolitics and refers to the phenomenon that certain parts of the population have strategic, economic and political advantage over other parts of the population and that those advantages are related to the geographical location and circumstances. In the field of ‘geomedicine’ the phrase it is related to the phenomenon that health care quality, access to health care and its utilization vary across regions. Where you live largely determines what health care you receive.

5 more details can be found in supplementary materials, textbox 1.

6 more details are given in supplementary materials, textbox 2.

7 details on these findings can be found in supplementary materials, textbox 3.

8 Beneficence, non-maleficence, autonomy, and justice – see textbox 2 in Supplementary Materials.

9 Nevertheless, we advise to continue to monitor and analyze the further spread of euthanasia across regions from this perspective (diffusion of new practices) in the years to come.

10 Preferably we would have included data on e.g. diagnoses, health factors at a patient level into our data set. Unfortunately no such data were available.

11 We would have preferred to select variables that are more directly indicative of people’s preferences. For example, a large-scale study has been conducted in the Netherlands in the past on citizens’ knowledge of, and attitudes toward, euthanasia [37, 38]. There is also a longitudinal national survey of the extent to which citizens support the option of euthanasia [39]. However, unfortunately these data were not reproducible, nor available at the level of postal codes or other regional indicators.

12 In the sections above we saw that Wennberg defines ‘preference-sensitive-care’ as: *procedures for which there is more than one option and where the outcomes may differ according to the option used. These services are called preference-sensitive because the decision as to which treatment is right for the individual patient should depend on a patient’s preference.*

13 Originally, J. Feinberg, who Schermer refers to, distinguishes between these two conceptions of autonomy, but we take them together here.

14 In 2021, 6 incapacitated patients received euthanasia in the Netherlands, 1.5% of euthanasia took place in patients with psychiatric diagnoses and 2.8% of euthanasia in patients with dementia [28].
