# Supplementary material for: Understanding regional variation in euthanasia using geomedical frameworks: a critical ethical reflection
Source: Res Health Serv Reg. 2023 Nov 29;2:19. doi: 10.1007/s43999-023-00034-6 (PMC11281760; doi:10.1007/s43999-023-00034-6)
Supplement: Supplementary file 1 — Additional file 1. [file 43999_2023_34_MOESM1_ESM.docx]

Supplementary materials – where you live is how you die

*Textbox 1 Three categories of care: different causes of (unwarranted) variation*

In an attempt to understand geographical variation in health care, Wennberg introduced a distinction between three categories of care, each with their own criteria for the (un)warrantedness of variation.[1-3]

Effective, or necessary care is defined as: *“those services that, on the basis of reasonably sound medical evidence, are known to work better than any alternative, and for which the benefits of treatment far exceed the side effects or unintended consequences. In other words, effective care includes any treatment that all eligible patients should receive, [based on] medical science, and objective, ‘high-quality’ information about the outcomes of treatment and evidence-based clinical guidelines that identify which patients stand to benefit.”* [3, p. 8] An example of effective care is the standard testing of newborns for phenylketonuria (PKU, an inborn and serious error of metabolism), which in most countries is now standard practice. [4] In contrast, ineffective or unnecessary care refers to treatments which, according to the latest scientific insights, should never be given to (certain) patients (under certain conditions). In 2016 Dutch health scientists provided a list of 1,366 ‘low value’ procedures (such as the use of FDG-PET in the detection of micro metastases), of which 77% should *never* be offered, regardless the circumstances. [5] Besides ‘ineffective,’ low value care can also be ‘unwanted’ or ‘inefficient.’ [6] In case of (in)effective care, appropriate care encompasses those services that are given precisely according to the evidence based guidelines (see figure 1). All variation implies a deviation from these guidelines, and is thus unwarranted (apart from cases in which well-informed patients would waive such care). In case of effective care, variation indicates underuse, whereas overuse is most certainly at stake in case of variation in ineffective care.

A second category of care that varies is elective, or ‘preference-sensitive’ care: “*procedures for which there is more than one option and where the outcomes may differ according to the option used. These services are called preference-sensitive because the decision as to which treatment is right for the individual patient should depend on a patient’s preference.”* [3, p.9] “*One example is the use of lumpectomy or mastectomy for treating early stage breast cancer. Another example is the treatment of early stage prostate cancer, for which the treatment options include surgery, various forms of radiation, and watchful waiting.”* [2, p.962] Because most patients delegate decision making to doctors, and because the evidence the for appropriate treatment is surrounded by a ‘grey zone’ (see the acceptable deviation from evidence based care in figure 1), part of the variation in the category of preference-sensitive care may be unwarranted. Wennberg argues: “*this can result in serious, (…) medical errors: operating on the wrong patients: those who, had they been fully informed, would not have wanted the operation they received.”* [3, p.10]

The third category is ‘supply-sensitive care.’ It is the type of care which frequency and intensity of use is highly determined by the medical professionals’ decisions, and also by the capacity and availability of care. Wennberg refers to care for acute and chronically ill patients, such as: *“physician visits, referrals for consultation, home health care, imaging exams and other diagnostic procedures, and admissions to hospitals, Intensive Care Units (ICUs), and skilled nursing homes.”* [3, p.11] For these procedures, Wennberg states, *“medical theory and medical evidence play virtually no role in determining the relative frequency of their use. For example, patients with chronic diseases such as diabetes, congestive heart failure, cancers, and obstructive pulmonary disease who live in regions with more doctors per capita will have more consultations and diagnostic tests (which are associated with consultations). If they live in regions with more beds they will experience more hospitalizations and stays in intensive care units.”* [2, p. 962] Thus; in supply-sensitive care it is a combination of the doctor’s decisions and the capacity of the ‘local medical market’ (the per capita numbers of available doctors, and hospital or ICU beds, for example) that strongly influences its utilization. Here again: insofar the health care provided deviates from what is necessary, according to the latest medical evidence, it is inappropriate and thus unwarranted (red circle in figure 1).

*Textbox 2 – Four principles of biomedical ethics (Beauchamp & Childress, 2013)*

1. Beauchamp and Childress discern *benevolence* – the character trait or virtue of being disposed to act for the benefit of others – from the principle of *beneficence*, which refers to a statement of moral obligation to act for the benefit of others. [Beauchamp & Childress, 2013, p. 203] The principle of beneficence supports an array of prima facie rules of obligation, including the following: protect and defend the rights of others; prevent harm from occurring to others; remove conditions that will cause harm to others; help persons with disabilities; rescue persons in danger. [ibid. p. 204]
2. In medical ethics the principle of *nonmaleficence* has been treated as effectively identical to the celebrated maxim *Primum non nocere:* “Above all (or first) do not harm.” Whereas the principle of beneficence refers to the (active) prevention and the removal of harm, and the promotion of good, nonmaleficence encompasses negative prohibitions of action: “*one ought not to inflict evil or harm.”* [ibid. p. 152] Rules that specify the principle of nonmaleficence are: do not kill, do not cause pain or suffering, do not incapacitate, do not cause offence, and do not deprive others of the goods of life. [ibid. p.154]
3. The word *autonomy* is derived from the Greek *autos* (‘self’) and *nomos* (‘rule,’ ‘governance,’ or ‘law’), and originally refers to the self-rule or self-governance of independent city-states. [ibid. p. 101] Beauchamp and Childress hold a ‘three-condition-account of autonomy, which requires *agency*, consisting of a) an intentional act of the individual; b) understanding of options and consequences; and c) *liberty*, or noncontrol. [ibid., p. 102-105] Also, it is important to distinguish between the negative and the positive obligation that comes forth from the principle of autonomy. As a negative obligation, the principle requires that autonomous actions not be subjected to controlling constraints by others. As a positive obligation, the principle requires both respectful treatment in disclosing information and actions that foster autonomous decision making. [ibid., p. 107]
4. The principle of *justice* is about what is due or owed to persons in terms of for example medical treatments at the EoL. Terms that have been used here, are *fairness, desert* (what is deserved) and *entitlement.* If resources are scarce, fair, equitable and appropriate distribution becomes a point of discussion. In the light of our subject (the geographical variation in the incidence of euthanasia), an important question is whether there are objectifiable differences between regions that justify the variations, or if variation is caused by factors that are no ground for moral justification and therefore cause infringements upon the principle of justice.

*Textbox 3 At a glance: geographical variation in the incidence of euthanasia in the Netherlands*

The incidence of euthanasia *in the Netherlands* as a percentage of all deaths – the euthanasia rate – increased during the past few decades: from an estimated 1.9% in 1990 [7, 8] to 4.5% in 2021.[9] Although this may seem a relatively low percentage at a national level, our study of euthanasia percentages (2013-2017) shows considerable variation between regions, municipalities, and districts within municipalities.[10] We shortly summarize the main insights and conclusions here, and refer to the original publication for more details.

The euthanasia rate of the three highest PC2 *regions* – the 89 2-digit postal code areas in the Netherlands – increased from 5.54 in 2013 to 6.88 in 2017; as euthanasia rates in the lowest regions increased even more, the practice variation decreases between 2013 and 2017. When omitting the outliers (‘P95 / P5’), the variation is smaller.

At the *municipal leve*l the euthanasia rates in the three highest-scoring municipalities are stable at 10%. The difference between the three municipalities with the most cases of euthanasia and the three municipalities with the fewest cases is a factor of 27 in 2013. A stronger increase in euthanasia rates in low-incidence municipalities leads to less variation over the years, to a factor of 17 in 2017. The number of municipalities with zero cases of euthanasia also decreased.

At the *district level* – a PC4-level – for the three districts with the highest euthanasia rates, the figures are considerably higher: around 16% of deaths there involve euthanasia. Districts without euthanasia are becoming more scarce (a decrease of 78% over five years: from 2,007 to 1,565 of the total of about 3,750 districts).

Finally, a comparison of *districts* *within* *major cities*. In Amsterdam, the percentage of euthanasia increased from 11.88% of total deaths in the three highest-incidence districts in 2013 to 14.43% in 2017. In Rotterdam, the numbers remained stable around 6% while in The Hague, the percentage in high-incidence neighbourhoods increased from 7.38% to 11.23%. While the variation in Amsterdam is increasing (7.2 in 2013 – 9.6 in 2017), an increasing number of cases of euthanasia in low-incidence neighbourhoods in Rotterdam and The Hague actually results in a decreasing variation (7.9 in 2013 –5.6 in 2017, respectively 9.2 in 2013 –5.2 in 2017).

Six variables are statistically significantly associated with the euthanasia incidence: (1) age: more residents 45-64 and a 3.4% higher euthanasia rate; (2) church attendance: more regular churchgoers and a 1.9% lower euthanasia rate; (3) voting patterns: more voting for a progressive party and a 0.9% higher euthanasia rate; (4) volunteers: more volunteers and a 1.3% lower euthanasia rate; (5) income: more better-off people and a 3.7% higher euthanasia rate; and finally (6) better perceived health and a (2.4%) higher euthanasia rate.

Over the years 2013-2017, the raw, unadjusted euthanasia rates show a difference between the highest versus the lowest three municipalities of a factor of 25.28. Adjusted for the six variables mentioned, an unexplained factor score of 10.30 remains.

References

1. Wennberg, J., K. McPherson, and D.C. Goodman, *Small Area Analysis and the Challenge of Practice Variation*, in *Medical Practice Variations*, A. Johnson and T.A. Stukel, Editors. 2016, Springer US: Boston, MA. p. 1-24.

2. Wennberg, J.E., *Unwarranted variations in healthcare delivery: implications for academic medical centres.* British Medical Journal, 2002. **325**(7370): p. 961-964.

3. Wennberg, J.E. and ProQuest (Firm), *Tracking medicine a researcher's quest to understand health care*. 2010, Oxford University Press,: Oxford. p. xviii, 319 p.

4. Wilcken, B., *Newborn Screening: Gaps in the Evidence.* Science, 2013. **342**(6155): p. 197-198.

5. Wammes, J.J., et al., *Identifying and prioritizing lower value services from Dutch specialist guidelines and a comparison with the UK do-not-do list.* BMC Med, 2016. **14**(1): p. 196.

6. Verkerk, E.W., et al., *Limit, lean or listen? A typology of low-value care that gives direction in de-implementation.* Int J Qual Health Care, 2018. **30**(9): p. 736-739.

7. Onwuteaka-Philipsen, B.D., et al., *Trends in end-of-life practices before and after the enactment of the euthanasia law in the Netherlands from 1990 to 2010: a repeated cross-sectional survey.* Lancet, 2012. **380**(9845): p. 908-15.

8. van der Heide, A., et al., *End-of-life practices in the Netherlands under the Euthanasia Act.* N Engl J Med, 2007. **356**(19): p. 1957-65.

9. RTE, *Jaarverslag 2021*, in *Regionale Toetsingscommissie Euthanasie. Jaarverslag*, euthanasiecommissie.nl, Editor. 2022: Den Haag.

10. Groenewoud, A.S., et al., *Euthanasia in the Netherlands: a claims data cross-sectional study of geographical variation.* BMJ Support Palliat Care, 2021.
