# Supplementary material for: Understanding regional variation in euthanasia using geomedical frameworks: a critical ethical reflection
Source: Res Health Serv Reg. 2023 Nov 29;2:19. doi: 10.1007/s43999-023-00034-6 (PMC11281760; doi:10.1007/s43999-023-00034-6)
Supplement: Supplementary file 2 — Additional file 2. [file 43999_2023_34_MOESM2_ESM.docx]

**Supplementary table 1**

|  | Euthanasia | Assisted suicide |
| --- | --- | --- |
| Australia (all six states) | * | * |
| Austria | - | * |
| Belgium | * | * |
| Canada | * | * |
| Colombia | * | - |
| Germany (awaiting regulation) | - | * |
| Luxembourg | * | * |
| the Netherlands | * | * |
| New Zealand | * | * |
| Portugal (awaiting regulation) | * | * |
| Spain | * | * |
| United States (California, District of Columbia, Colorado, Hawaii, Maine, Montana, New Jersey, Oregon, Vermont, Washington) | - | * |
| Switzerland | - | * |

* legal / - not legal
